# Effectiveness of oral mucositis management based on MASCC/ISOO guidelines in pediatric oncology patients: a randomized controlled trial

**DOI:** 10.1007/s00520-026-10393-8

**Published:** 2026-03-21

**Authors:** Zübeyde Ezgi Erçelik, Dilek Bayram, Birgül Erdoğan, Naime Altay

**Affiliations:** 1 Faculty of Health Sciences, Department of Nursing, Division of Pediatric Nursing, Bandırma Onyedi Eylül University, Balıkesir, Türkiye; 2 Training and Research Hospital, Pediatric Oncology Clinic, Zonguldak Bülent Ecevit University, Zonguldak, Türkiye; 3 Faculty of Health Sciences, Department of Nursing, Division of Pediatric Nursing, Kocaeli University, Kocaeli, Türkiye; 4 Nursing Faculty, Division of Pediatric Nursing, Gazi University, Ankara, Türkiye

**Keywords:** Childhood cancer, Oral mucositis, Oral care, Chemotherapy, Pediatric oncology

## Abstract

**Purpose:**

Although survival rates in childhood cancer have improved with more effective, risk-adapted multimodal treatments, oral mucositis remains one of the most common treatment-related oral complications. This study evaluated the impact of an oral mucositis care protocol on the severity of mucositis.

**Methods:**

This prospective randomized controlled study was conducted with 30 pediatric oncology patients aged 6 to 18 years. The sample included children with hematologic malignancies (predominantly acute lymphoblastic leukemia) and solid tumors. The data were collected using a descriptive information form, the Children’s International Mucositis Evaluation Scale (ChIMES), and the WHO Oral Mucositis Grading Scale. Standard care was applied to the control group. For the intervention group, an oral care protocol was prepared, and the patients received training. The oral care intervention was applied for 14 days and monitored by the researcher. The patients were monitored for oral mucositis on Days 0, 3, 7, and 14. Those in the intervention group received a calendar for documenting their oral care practices.

**Results:**

The ChIMES scores showed a significant difference between the groups and were lower in the intervention group (*z* = 0.010; *p* = 0.011). The WHO scale results revealed significant differences between the groups on Day 7 (*Z* = −3.106; *p* = 0.002) and Day 14 (*Z* = −2.841; *p* = 0.005).

**Conclusion:**

At the end of the study, the severity of mucositis was lower in the patients who received the oral mucositis care protocol. It is recommended that a standardized oral care protocol specific to children be developed and that education on oral mucositis care be provided to children and their parents from the beginning of hospitalization.

**Clinical trial registration:**

ClinicalTrials.gov (Identifier: NCT06711315; Registration date: 29 November 2024).

## Introduction

In recent years, the incidence of childhood cancers has increased significantly throughout the world and in Türkiye. According to the IARC Global Cancer Observatory report, more than 280,000 children and adolescents (0–19 years) were diagnosed with cancer worldwide in [1]. Chemotherapy is one of the most commonly used treatment methods in children diagnosed with cancer [2]. Despite continuous advancements in childhood cancer treatments, oral mucositis (OM) affects approximately 52–80% of children undergoing cancer therapy [3].

Mucositis is an acute side effect seen as ulcerative lesions in the oral and/or gastrointestinal tracts due to epithelial tissue damage [4]. It typically develops 3–10 days following the initiation of chemotherapy, with its peak incidence occurring around 7–14 days post-treatment. Clinical findings can range from mild pain and erythema to severe ulcerations [5]. OM can lead to difficulty with chewing and/or swallowing, weight loss, inflammation, edema, impaired oral intake, and, in some cases, the need for parenteral nutrition in children [6]. These complications may cause treatment delays, interruptions, or dosage decreases, which result in increased healthcare costs, mortality, and morbidity and significantly reduced quality of life [2, 7].

To effectively manage OM, comprehensive protocols should be developed before starting chemotherapy treatment [8]. Existing protocols and studies indicate that various oral care practices are utilized to prevent OM in pediatric patients [8–10]. However, the characteristics of the solutions used in these studies and the duration and frequency of use vary. Additionally, there is insufficient data regarding the use of certain solutions in pediatric populations. The 2020 guide published by the Multinational Association of Supportive Care in Cancer and the International Society of Oral Oncology (MASCC/ISOO) emphasizes that the primary strategies for preventing OM include oral care protocols and patient education [6]. These guidelines support multi-component oral care approaches (Level of evidence III)—including brushing with a soft toothbrush, flossing, and rinsing with saline and sodium bicarbonate—as part of routine care, while noting that evidence for some agents (e.g., oral glutamine) is limited and largely based on specific patient populations rather than routine prophylaxis in children. In addition, mucositis assessment using appropriate OM scales and the use of written and visual educational materials are recommended as key components of OM management [4, 11]. Recent work published in EClinicalMedicine has also underscored the importance of using standardized, validated tools for mucositis assessment in both clinical practice and research [10]. High-quality, effective oral hygiene plays a critical role in the prevention of mucositis and associated infections. It has been reported that when OM protocols are followed regularly, there is a decrease in the incidence and severity of OM [9].

While many protocols have been used to prevent OM, pediatric oncology nurses bear the primary responsibility for OM care and prevention. However, most of these recommendations are for adult patients, and data regarding pediatric populations remain limited. There is a need for evidence-based data specifically addressing OM prevention and management in pediatric patients. This study aims to contribute to this gap in pediatric oncology care by evaluating the impact of OM protocols on the incidence and severity of OM in children.

## Method

### Study design

The study was conducted using a randomized controlled experimental study design with children who were undergoing chemotherapy treatment in the oncology clinic of a training and research hospital.

### Setting

The study was conducted between July 20, 2023, and October 20, 2024. This study was conducted in a Training and Research Hospital in Türkiye.

### Sample size and participants

**Fig. 1:**
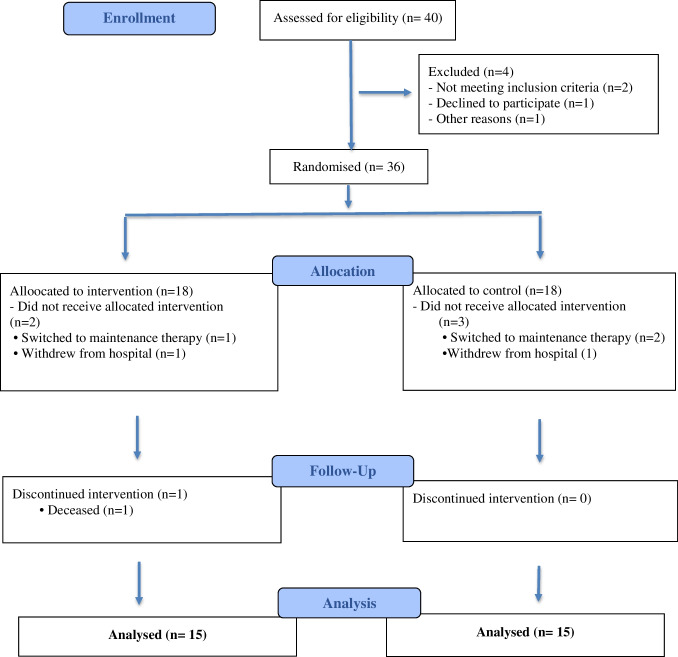
CONSORT flowchart

The population of the research consisted of children pediatric cancer patients hospitalized in the oncology clinic of the hospital. G*Power-3.1.9.7 program was used to calculate the sample and power. While calculating the required sample size for our study, we referenced the similar study of Kostak et al. (2020). Accordingly, it was determined that a total of 30 children, 15 children in each group, should be included in the study for a power of 90% at a Type I error level of 5%. Assuming a loss of 20% in each group during the intervention, 18 children were included in the study. However, we could not include two children in the intervention group, and one child did not want to answer the post-test survey questions. Similarly, three children from the control group withdrew from the study. The study was completed with 15 children in the intervention and the control group (Fig. 1).

Inclusion criteria were being between 6 and 18 years, having received at least 1 course of chemotherapy, having no visual, auditory, or intellectual problems, speaking and understanding Turkish, and being willing to participate in the research. Additional inclusion criteria were having no pre-existing oral mucosal lesions, no history of chronic dental or oral disease, and no previously documented severe oral mucositis related to prior chemotherapy cycles.

Exclusion criteria were having a problem that prevents verbal communication (neurodevelopmental retardation, verbal speech difficulties, hearing or hearing problems, having oral mucositis). In addition, children who were receiving any antiviral or antifungal therapy specifically prescribed for oral mucositis at baseline were excluded from the study.

### Randomization

After obtaining ethical approval from the university ethics committee and the hospital and obtaining approval from the parents of children with cancer between the ages of 6 and 18 years, randomization was performed among the children who volunteered to participate in the study. To ensure equal distribution of participants between the groups, the assignment to the intervention and control groups was carried out using a closed envelope system. The researchers instructed the nurse responsible for the clinic to draw the papers from the envelope (lottery method) using the blinding technique. The group numbers were sequentially assigned to the intervention and control groups in a 1:1 ratio. The random allocation sequence was generated using a computer-based random number generator (randomizer.org) by a researcher who was not involved in participant recruitment or data collection. Sequentially numbered, opaque, sealed envelopes containing group assignments were prepared to maintain allocation concealment. During participant enrollment, the nurse responsible for the clinic opened the next envelope in sequence to assign each participant to the intervention or control group in a 1:1 ratio. The group allocation was concealed from the data analysts, and all data were analyzed by an independent statistician blinded to group assignments. The data were analyzed by an independent statistician who was unaware of the intervention and control groups.

In this study, participant and researcher blinding was not implemented. Blinding was impossible because the researcher monitored participants and their parents on a daily basis and was aware of the intervention.

### Data collection tools

In the study, the data were collected using the Descriptive Information Form, Children’s International Mucositis Evaluation Scale (ChIMES), and the WHO Oral Mucositis Grading Scale. The data were collected by the researcher through the face-to-face interview technique.

### Descriptive information form

The introductory information form is a brief questionnaire that collects basic information about the parents and child. It was developed by the researchers according to the literature [12, 13] and includes questions regarding the child’s age, gender, diagnosis, parental information, duration of chemotherapy, and frequency of tooth brushing. Baseline oral health status (presence of oral lesions, history of dental or oral problems, and previous severe oral mucositis episodes) was also recorded using this form.

### Children’s International Mucositis Evaluation Scale (ChIMES)

ChIMES was developed by Tomlinson et al. [14] to evaluate the development of oral mucositis in children. The scale consists of categories such as the severity of pain in the mouth, the effect of pain on swallowing, eating, drinking, taking painkillers, and the presence of ulcers in the mouth. The lowest score obtained from the scale is 0, and the highest score is 23. The higher the total score, the more advanced the degree of mucositis [12, 14]. Cronbach alpha reliability coefficient of the scale is 0.91. In the study, the Cronbach’s alpha (α) reliability coefficient was found to be 0.76 for the total scale.

### WHO Oral Mucositis Grading Scale

The presence of oral mucositis is assessed based on clinical manifestations. The score ranges from 0 (the absence of manifestations and symptoms) to 4 (oral feeding is impossible) [15]: Grade 0, no change in mucosa; Grade 1, painless ulcers, erythema, or mild sensitization; Grade 2, painful erythema and ulcer but can consume solid foods; Grade 3, painful erythema, edema, or ulcer but can consume only fluids; and Grade 4, ulceration, necrosis, and hemorrhage, patient cannot feed, and enteral or parenteral support is required. In the study, the Cronbach’s alpha (α) reliability coefficient was found to be 0.84 for the total scale.

### Data collection process

The researchers informed children and their parents about the purpose of the study. Written and verbal consent was obtained from both willing children and their parents.

The researcher works as a nurse in the pediatric oncology clinic. If the children met the inclusion criteria, the researchers visited the clinics, introduced themselves to the child and the family, and provided information about the research. The child and the family were informed about the research. All caregivers (both intervention and control groups) who meet the inclusion criteria and agree to participate in the study will be asked to fill out the Descriptive Characteristics Form. Research data will be obtained by conducting four assessment interviews with each child and caregiver. The first interview will be conducted before the treatment, the second interview will be conducted on the 3rd day after the treatment, and the following interviews will be conducted on the 7th and 14th days of the treatment, and the oral mucosa will be evaluated by the researcher by interviewing the child and caregiver and using scales.

The child and caregiver in the intervention group will receive an oral care protocol on days 0–14 of treatment. The child and caregiver in the control group will receive standard care. To enhance intervention fidelity and standardization of assessment, all evaluations were conducted by the same nurse researcher using a standardized assessment protocol with the ChIMES and WHO scales at each time point. In the intervention group, adherence to the oral care protocol was monitored daily by reviewing the oral care calendar together with the child and caregiver. The independent statistician who analyzed the data was blinded to group allocation.

### Procedures

#### Intervention group

The children in the intervention group were subjected to a four-stage intervention carried out by the nurse researcher. The practices in each stage were as follows:

- Stage 1: Oral care protocol training to prevent oral mucositis.
- Stage 2: Introduction and application of training material (brochure).
- Stage 3: Introduction and implementation of oral care calendar.
- Stage 4: Implementation of oral care protocol.

##### Stage 1: Oral care protocol training to prevent oral mucositis

An oral care protocol was developed to standardize oral care practices for children and caregivers prior to chemotherapy treatment. The protocol was informed by MASCC/ISOO 2020 clinical practice guidelines for basic oral care (e.g., gentle toothbrushing and saline/sodium bicarbonate rinses) and complemented by the existing institutional oral care protocol used in the pediatric oncology clinic. Expert opinions were gathered to ensure the content of the training was comprehensive. The training session, conducted in a single session, utilized a PowerPoint presentation that covered key aspects such as the definition of oral mucositis, its etiology, signs and symptoms, and the details of the oral care protocol. Following the presentation, the researcher provided one-on-one guidance with each child and their caregiver, ensuring that they understood and could apply the oral care protocol. During this session, the researcher demonstrated the application of the protocol and addressed any questions. All materials to be used in the protocol were introduced individually to each patient. At the conclusion of the training, participants were given a brochure containing the materials, instructions, and application steps for the protocol. Separate training sessions were conducted for each child hospitalized in the clinic, along with their caregiver.

##### Stage 2: Introduction and application of training material (brochure)

**Fig. 2:**
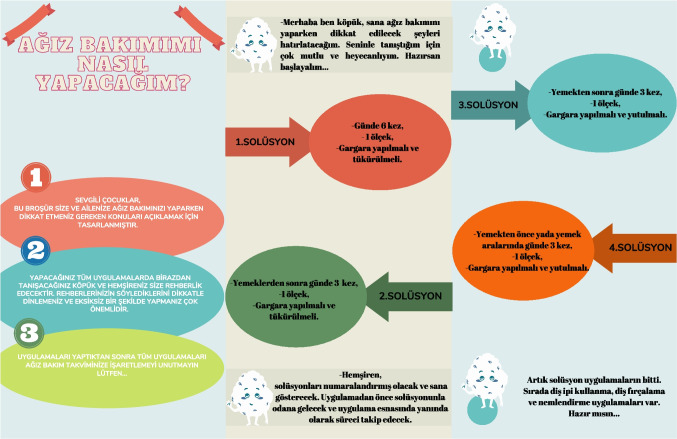

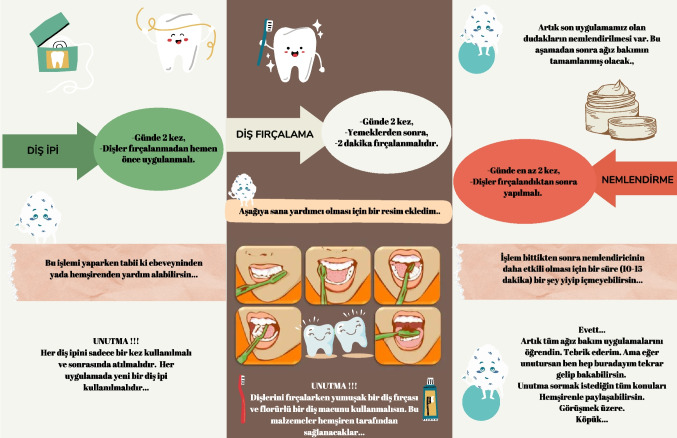
Brochure

Each child received a brochure that included detailed instructions on the use of the prescribed oral care products, including the recommended frequency of use per day. The brochure served as a practical guide for both the child and caregiver to ensure proper application and adherence to the protocol (Fig. 2).

##### Stage 3: Introduction and implementation of oral care calendar

**Fig. 3:**
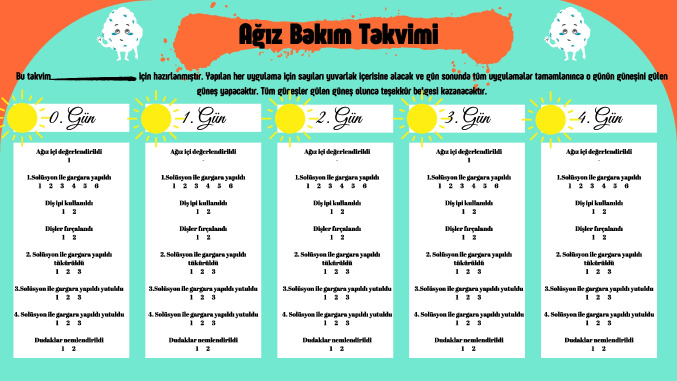

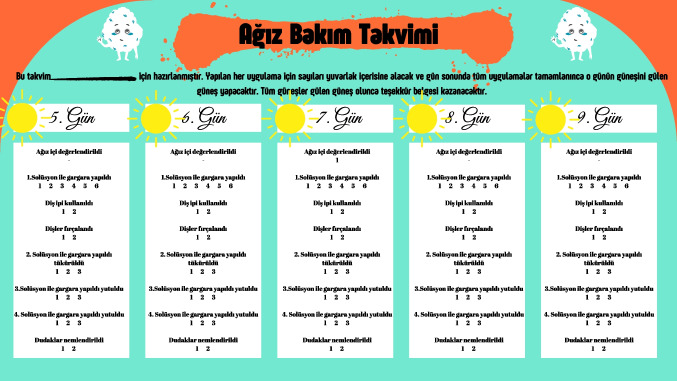

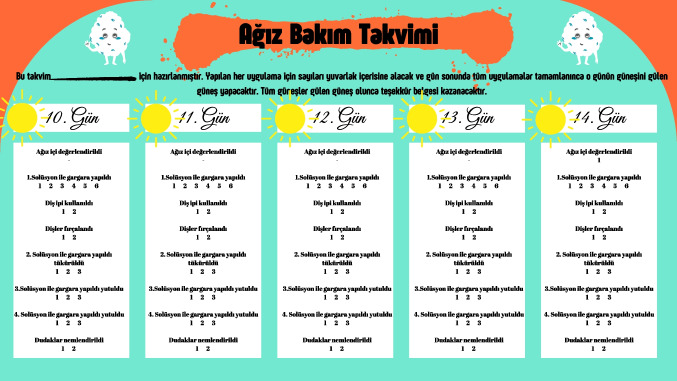
Followed oral care calendar and training

Patients in the intervention group were provided with a calendar to document their oral care practices. This calendar allowed the child, their caregiver, and the nurse to monitor and track oral care practices over time (Fig. 3).

**Fig. 4:**
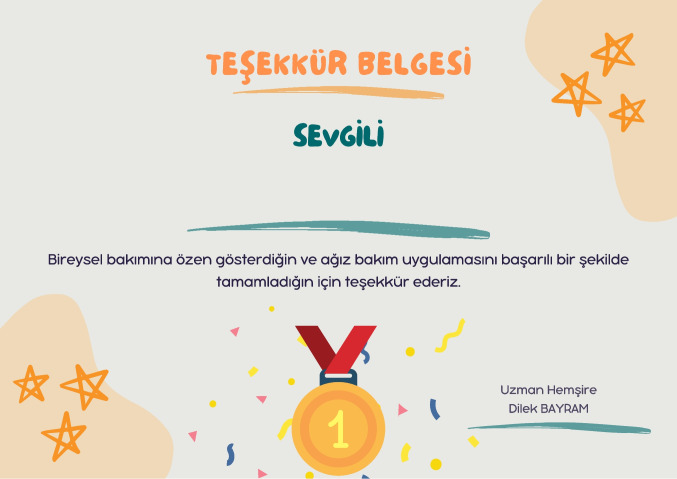
Certificate

Children in the intervention group who adhered to and completed the oral care protocol for 14 days were awarded a certificate of appreciation, which was prepared by the researchers (Fig. 4).

##### Stage 4: Implementation of oral care protocol

Within the scope of the study, an oral care protocol, developed based on the existing literature, MASCC/ISOO oral mucositis management recommendations for basic oral care, and the institutional oral care protocol, was applied to the intervention group over a 14-day period. The selection and concentration of products (sodium bicarbonate, sodium chloride solution, glutamine, lanolin-containing lip moisturizer, and Mycostatin) were based on published recommendations for oral care and on the pediatric oncology clinic’s long-standing institutional practices. Glutamine and Mycostatin were included as part of this institutional protocol rather than as agents specifically recommended in the MASCC/ISOO guideline for routine prophylaxis in pediatric patients. The frequency of rinsing and applications was determined to provide regular mechanical and chemical cleansing while remaining feasible for children and caregivers in the inpatient setting. Laboratory values were monitored, and the application was evaluated during the application process.

Prior to treatment (Day 0), each child in the intervention group underwent an oral examination. Based on the results of the evaluation, participants were instructed to gargle with sodium bicarbonate (5 cc) six times per day starting on Day 1 and to gargle and spit between meals. Additionally, they were provided with mouthwash containing sodium chloride (5 cc), which they were instructed to spit out, and Mycostatin (2 cc), which was to be swallowed after each meal. Participants were also asked to gargle and swallow glutamine (5 cc) three times a day, either before or between meals.

Finally, participants were instructed to apply a Lanolin-containing lip moisturizer at least twice daily and to refrain from eating or drinking for 30 min after application. Table 1 outlines the daily oral care protocol for the intervention group. Evaluations were conducted on Days 0, 3, 7, and 14 of the intervention.

**Table 1:** The daily oral care practice

Treatment method	Administration method	Day 0	Day 1	Day 2	Day 3	Day 4	Day 5	Day 6	Day 7	Day 8	Day 9	Day 10	Day 11	Day 12	Day 13	Day 14
Sodium bicarbonate (5 cc)	Rinse and spit		**6 × 1**	**6 × 1**	**6 × 1**	**6 × 1**	**6 × 1**	**6 × 1**	**6 × 1**	**6 × 1**	**6 × 1**	**6 × 1**	**6 × 1**	**6 × 1**	**6 × 1**	**6 × 1**
Sodium chloride (5 cc)	Rinse and spit		**3 × 1**	**3 × 1**	**3 × 1**	**3 × 1**	**3 × 1**	**3 × 1**	**3 × 1**	**3 × 1**	**3 × 1**	**3 × 1**	**3 × 1**	**3 × 1**	**3 × 1**	**3 × 1**
Glutamine (2 cc)	Rinse and swallow		**3 × 1**	**3 × 1**	**3 × 1**	**3 × 1**	**3 × 1**	**3 × 1**	**3 × 1**	**3 × 1**	**3 × 1**	**3 × 1**	**3 × 1**	**3 × 1**	**3 × 1**	**3 × 1**
Glutamine (5 cc)	Rinse and swallow		**3 × 1**	**3 × 1**	**3 × 1**	**3 × 1**	**3 × 1**	**3 × 1**	**3 × 1**	**3 × 1**	**3 × 1**	**3 × 1**	**3 × 1**	**3 × 1**	**3 × 1**	**3 × 1**
*Dental floss	To be used before brushing teeth		**2 × 1**	**2 × 1**	**2 × 1**	**2 × 1**	**2 × 1**	**2 × 1**	**2 × 1**	**2 × 1**	**2 × 1**	**2 × 1**	**2 × 1**	**2 × 1**	**2 × 1**	**2 × 1**
*Soft toothbrush	To be used after morning and evening meals		**2 × 1**	**2 × 1**	**2 × 1**	**2 × 1**	**2 × 1**	**2 × 1**	**2 × 1**	**2 × 1**	**2 × 1**	**2 × 1**	**2 × 1**	**2 × 1**	**2 × 1**	**2 × 1**
Lanolin-based moisturizer	To be applied after tooth brushing		**2 × 1**	**2 × 1**	**2 × 1**	**2 × 1**	**2 × 1**	**2 × 1**	**2 × 1**	**2 × 1**	**2 × 1**	**2 × 1**	**2 × 1**	**2 × 1**	**2 × 1**	**2 × 1**
Oral cavity assessment		**1 × 1**			**1 × 1**				**1 × 1**							**1 × 1**

*All applications and assessments will be conducted by the research nurse. Solution dosages and applications will be adjusted based on laboratory assessments and oral cavity evaluations. Dosage increases or exclusions may be made if necessary (platelet levels will be monitored for dental brushing and flossing procedures)

### Control group

Children in the control group will receive standard care, which includes mouthwash with sodium bicarbonate after each feeding, Mycostatin (2 × 1), and Tranflex (3 × 1). This standard oral care protocol reflects the routine practice of the hospital and includes active components such as sodium bicarbonate mouthwash, Mycostatin, and Tranflex. No additional mucositis-specific interventions (e.g., glutamine supplementation, cryotherapy, or photobiomodulation therapy) were permitted in the control group during the study. The use of a toothbrush and dental floss was not systematically encouraged as part of the standard protocol for this group. At the end of the 14-day period, both the child and their caregiver in the control group will receive oral care protocol training to prevent oral mucositis.

### Data analysis

The licensed Statistical Guideline for the Social Sciences (SPSS) for Windows IBM version 29 guideline program was used for data analysis. Descriptive statistics were presented using minimum-maximum, frequency (*n*), and percentage (%) values for categorical variables and mean ± standard deviation (X ± SD) values for continuous variables. The chi-square test was used to compare categorical variables according to groups. The normality of continuous variables was analyzed with the Shapiro-Wilk test. Pearson Chi-squared (χ2) test, Wilcoxon test, and Mann-Whitney *U* test were used to analyze the data. Because the main outcome variables (ChIMES and WHO Oral Mucositis Grading Scale scores) were non-normally distributed and measured repeatedly at four time points (Days 0, 3, 7, and 14), within-group changes over time were analyzed using the Friedman test. Between-group comparisons at each time point were performed using the Mann-Whitney *U* test. The results were evaluated at a 95% confidence interval, and the significance level was set at *p* < 0.05.

## Results

The research discussed here presents findings from 30 participants. At baseline, before comparing the outcomes at follow-up, factors such as child’s sex, the age of the mother, father, and child, the mother’s and the father’s educational status, income status, chemotherapy duration, and tooth brushing habits were examined. The baseline characteristics were similar between the intervention group and the control group. Specifically, 53.3% were between the ages of 10–14 in the intervention group, and 66.7% were between the ages of 10–14 in the control group. Most of the participants were girls in both groups, 60% and 53.3%, respectively. Girls comprised 84% (*n* = 63) of the children, while boys accounted for 16% (*n* = 12). In the intervention group, 46.7% of the children had received chemotherapy for 4–6 months; 40% of the children in the control group had received chemotherapy for 7–9 months. It was found that 33.3% of the children in the e group and 26.7% of the children in the control group brushed their teeth once a day. Other descriptive characteristics are presented in Table 2.

**Table 2:** Findings about descriptive characteristics and the homogeneity of the groups

Variables	Intervention group (*n* = 15)		Control group (*n* = 15)		Statistical analysis
	*n*	**%**	*n*	*%*	
Gender					
Male	9	40	7	46.7	X^2^ = 0.536 *p* = 0.464
Female	6	60	8	53.3	
Age					
6–9	4	26.7	5	33.3	X^2^ = 3.33 *p* = 0.189
10–14	8	53.3	10	66.7	
15–18	3	20	-	-	
Age of mother					
26–35	4	26.7	3	20	X^2^ = 3.94 *p* = 0.139
36–45	8	53.3	12	80	
45 and above	3	20	-	-	
Mother’s education level					
Primary School	3	20	1	6.7	X^2^ = 3.990 *p* = 0.136
High School	8	53.3	13	86.7	
University	4	26.7	1	6.7	
Age of father					
26–35	2	13.3	2	13.3	X^2^ = 3.391 *p* = 0.183
36–45	10	66.7	12	86.7	
45 and above	3	20	-	-	
Mother’s education level					
Primary School	1	6.6	2	13.3	X^2^ = 0.000 *p* = 0.650
High School	9	60	8	53.4	
University	5	33.4	5	33.3	
Income status					
Less than my expenses	12	80	14	93.3	X^2^ = 1.154 *p* = 0.283
Equal to my expenses	3	20	1	6.7	
Diagnosis					
Leukemia	4	26.7	4	26.7	X^2^ = 4.500 *p* = 0.480
CNS tumor	2	13.3	2	13.3	
Lymphoma	5	33.3	3	20	
Sarcoma	-	-	3	20	
Neuroblastoma	3	20.6	3	20	
Wilms tumor	1	6.7	-	-	
Chemotherapy duration					
4–6 months	7	46.7	1	6,7	X^2^ = 9.700 *p*** = 0.021**
7–9 months	6	40	6	40,0	
10–12 months	-	-	5	33,3	
1 year and above	2	13.3	3	20	
Frequency of brushing teeth					
Not brushing	4	26.7	2	13,3	X^2^ = 4.778 *p* = 0.311
Once a day	5	33.3	4	26,7	
Twice a day	3	20		6,7	
Three times a day	-	-	1	13,3	
Irregular	3	20	6	40	

χ^2^ = Chi-square test

The comparison of CHIMES scale levels of intervention and control groups is given in Table 3. No significant difference was found between the groups at baseline (Day 0), Day 3, and Day 7 (*p* > 0.05). On day 14, there was a difference between the intervention group (5.26 ± 1.66) and the control group (6.86 ± 1.50) in terms of CHIMES scale total mean scores (*z* = 0.010; *p* = 0.011).

**Table 3:** Comparison of children’s CHIMES scores by groups and times (*n* = 30)

	Intervention group (*n* = 15)		Control group (*n* = 15)				
	x̄ ± SS	M (min–max)	x̄ ± SS	M (min–max)	z^1^	*p*	Friedman test *p*-value
Day 0	4.86 ± 2.19	4 (3–12)	5.20 ± 1.37	5 (4–8)	− 1.425	0.202	40.549 < **0.000**
Day 3	6.73 ± 1.75	6 (4–10)	7.13 ± 1.59	7 (5–10)	− 0.677	0.512	
Day 7	6.93 ± 1.48	7 (4–10)	8 ± 1.69	9 (5–10)	− 1.728	0.089	
Day 14	5.26 ± 1.66	5 (2–8)	6.86 ± 1.50	7 (3–10)	0.010	**0.011**	
Pairwise comparisons	t^2^	*p*	z^2^	*p*			
Day 0–Day 3	− 2.996	**0.030**	− 3.095	**0.002**			
Day 0–Day 7	− 2.574	**0.010**	− 3.441	**0.001**			
Day 0–Day 14	− 0.714	0.475	− 2.723	**0.006**			
Day 3–Day 7	− 0.537	0.591	− 2.289	**0.22**			
Day 3–Day 14	− 2.121	**0.034**	− 0.733	0.463			
Day 7–Day 14	− 2.394	**0.017**	− 2.382	**0.017**			

z^1^: Mann**-**Whitney*U*

Z^2^: Wilcoxon test

**Fig. 5:**
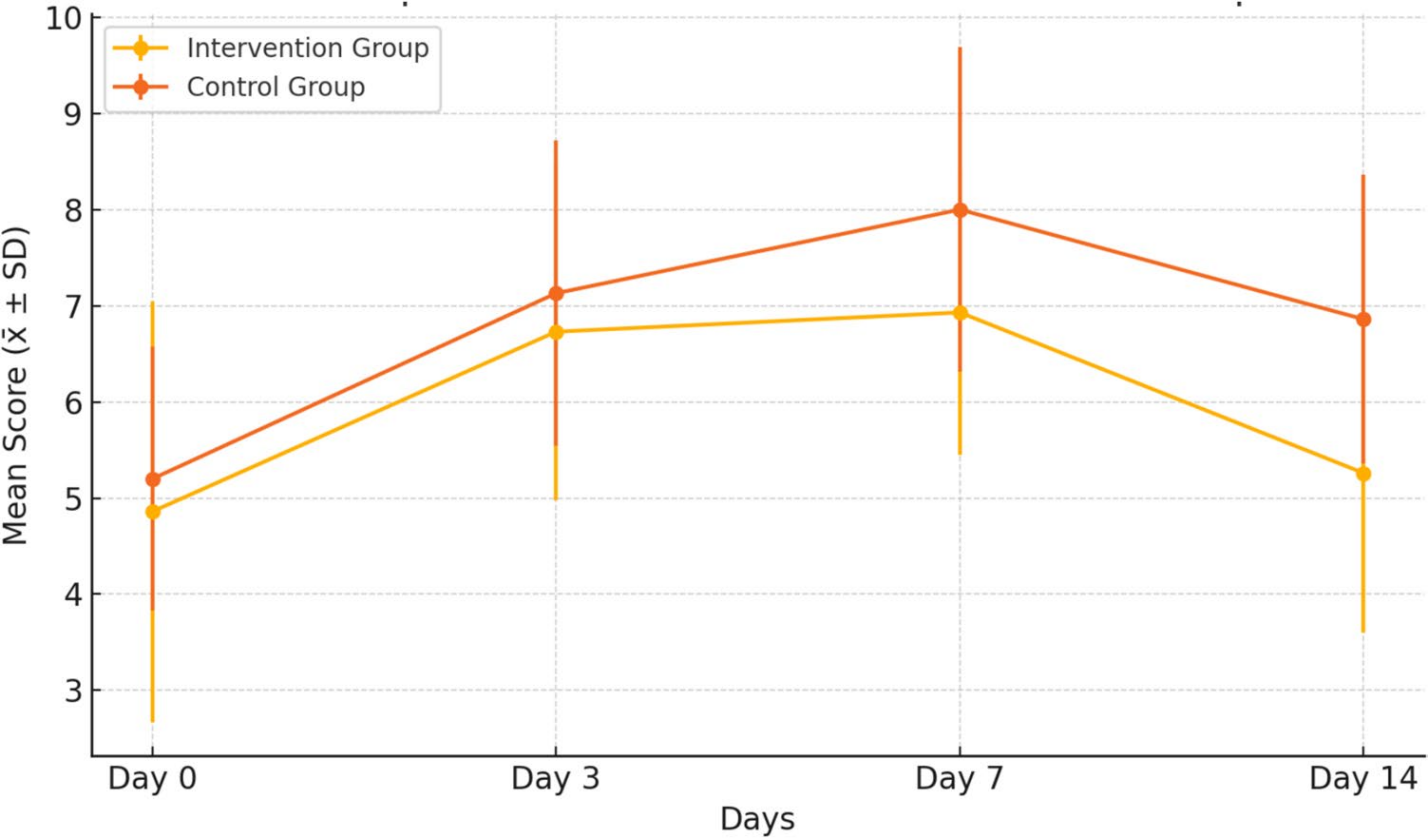
Changes in ChIMES scores over time in the intervention and control groups

There was a statistically significant difference in the within-group evaluation of ChIMES median scores across the four measurement time points in both the intervention and control groups. Pairwise comparisons showed that, in the intervention group, ChIMES scores on Day 14 were significantly lower than those on Days 3 and 7, indicating an improvement in mucositis severity over time. In contrast, in the control group, ChIMES scores remained relatively higher across the follow-up assessments. Findings from the Friedman test for the overall sample showed that there was a statistically significant difference in ChIMES scores across the four measurement time points (χ^2^ = 40.549, df = 3, *p* < 0.001). The mean rank scores indicated that the severity of mucositis increased progressively from the pre-test (mean rank = 1.40) to Day 7 (mean rank = 3.33), followed by a decrease on Day 14 (mean rank = 2.38). The changes in ChIMES scores over time for both groups are also illustrated in Fig. 5, which shows a greater reduction in mucositis severity in the intervention group compared with the control group, particularly between Days 7 and 14.

The comparison of WHO Oral Mucositis Grading Scale levels between the intervention and control groups is presented in Table 4. No significant difference was found between the groups at baseline (Day 0) or on Day 3 (*p* > 0.05). When the WHO scale medians were compared, the median score on Day 7 was 1 in the intervention group and 2 in the control group (*Z* = −3.106, *p* = 0.002). On Day 14, the median score was 1 in the intervention group (min 0, max 1) and 2 in the control group (min 0, max 2), and this difference was also statistically significant (*Z* = −2.841, *p* = 0.005).

**Table 4:** Comparison of children’s WHO Oral Mucositis Grading Scale scores by groups and times (*n* = 30)

	Intervention group (*n* = 15)		Control group (*n* = 15)				
	x̄ ± SS	M (min–max)	x̄ ± SS	M (min–max)	z^1^	*p*	Friedman test *p*-value
Day 0	0.40 ± 0.32	0 (0–2)	0.40 ± 0.50	1 (0–1)	− 1.207	0.227	63.423 < **0.000**
Day 3	1.13 ± 0.83	1 (0–3)	1.46 ± 0.51	1 (1–2)	− 1.425	0.153	
Day 7	1.53 ±.063	1 (1–3)	2.40 ± 0.63	2 (1–3)	− 3.106	**0.002**	
Day 14	0.60 ± 0.50	1 (0–1)	1.26 ± 0.59	1 (0–2)	− 2.841	**0.005**	
Pairwise comparisons	t^2^	*p*	z^2^	*p*			
Day 0–Day 3	− 3.051	**0.002**	− 3.35	**0.001**			
Day 0–Day 7	− 3.494	**0.000**	− 3.48	**0.000**			
Day 0–Day 14	− 1.134	0.257	− 2.88	**0.004**			
Day 3–Day 7	− 2.449	**0.014**	− 3.50	**0.000**			
Day 3–Day 14	− 2.138	**0.033**	− 1.34	0.180			
Day 7–Day 14	− 3.276	**0.001**	− 3.49	**0.000**			

z^1^: Mann**-**Whitney *U*

Z^2^: Wilcoxon test

**Fig. 6:**
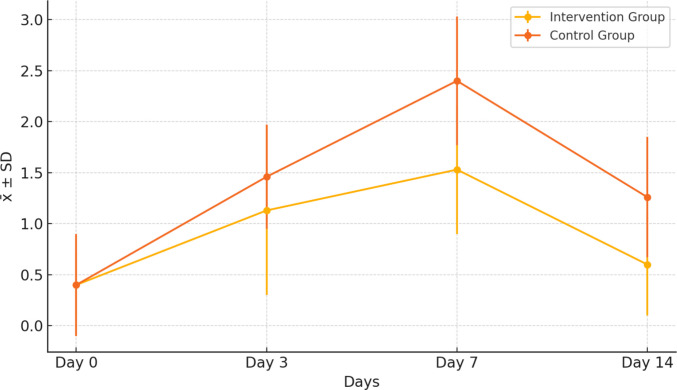
Changes in WHO Oral Mucositis Grading Scale scores over time in the intervention and control groups

There was a statistically significant difference in within-group WHO Oral Mucositis Grading Scale medians across the four time points in both the intervention and control groups. Findings from the Friedman test showed a statistically significant difference in mucositis scores across time (χ^2^ = 63.423, df = 3, *p* < 0.001). The mean rank scores indicated that the severity of mucositis increased progressively from Day 0 (mean rank = 1.45) to Day 7 (mean rank = 3.73), followed by a decrease at Day 14 (mean rank = 2.10). Figure 6 presents the trajectory of WHO Oral Mucositis Grading Scale scores over time, demonstrating lower mucositis grades in the intervention group than in the control group on Days 7 and 14.

## Discussion

Oral mucositis is among the most frequently encountered adverse effects of chemotherapy in pediatric oncology patients. Effective management of oral mucositis is essential to enhance the quality of life for affected children and to ensure high-quality nursing care. The prevention and management of oral mucositis in pediatric oncology requires a multifaceted approach integrating pharmacological and non-pharmacological interventions. However, there is limited evidence regarding the optimal application frequency and methods for both pharmacological and non-pharmacological strategies in pediatric settings. While some clinics employ individualized approaches to oral mucositis management, there remains a critical need for standardized, evidence-based oral care protocols.

A study conducted in Turkey by Arpaci et al. reported that more than half of pediatric oncology clinics lacked a written oral care protocol aligned with clinical guidelines [16]. Standardized, evidence-based oral care protocols are fundamental to the prevention and management of OM in pediatric oncology [17]. Several studies, along with MASCC/ISOO guidelines, emphasize the importance of implementing structured oral care protocols for the prevention and management of OM in pediatric patients [17–19]. According to MASCC/ISOO Clinical Practice Guidelines, multiagent combination oral care protocols—encompassing oral hygiene, topical and systemic treatments, and patient education—may reduce the incidence and severity of OM in children undergoing chemotherapy or stem cell transplantation [20].

In the present study, all oral hygiene and topical applications—including sodium bicarbonate, sodium chloride, glutamine, dental floss, soft toothbrushes, and lip balm—were selected based on recommendations from the literature and were informed by MASCC/ISOO oral mucositis management guidelines for basic oral care while also incorporating elements of the existing institutional oral care protocol (such as the use of oral glutamine), which are not specifically recommended for routine prophylaxis in pediatric patients [6, 8]. Although existing studies report positive findings regarding these practices, further research is needed to strengthen the evidence, particularly in pediatric populations.

The findings of the present study demonstrated a significant difference between the intervention and control groups in terms of total ChIMES scores on Day 14. While no differences were observed in the initial onset of OM (Days 0–3) according to the WHO Oral Mucositis Grading Scale, a significant difference was identified on Days 7 and 14. The literature indicates that OM typically manifests 3 to 10 days after the initiation of chemotherapy and peaks between Days 7 and 14 [8, 21–23]. Considering this trajectory, the intervention appeared to be most effective at the stage when OM severity peaks. Pairwise comparisons using both assessment scales revealed significant differences, particularly between Days 7 and 14. These findings suggest that the effectiveness of the intervention may be attributed to both the substances used and the frequency of application.

MASCC/ISOO also highlights patient education as a critical component of OM management. Given that this study was conducted in a pediatric population, both the child and their caregiver were actively involved in the process. The literature suggests that oral health education programs improve patient adherence and oral health outcomes, leading to a reduction in OM incidence, despite cultural variations [24, 25]. In this study, caregivers received *Oral Care Guideline Training to Prevent Oral Mucositis* prior to chemotherapy, with detailed explanations of the application process. This approach not only enhanced caregivers’ knowledge but also facilitated their active participation in oral care practices.

As part of the study protocol, both children and caregivers were responsible for implementing oral care practices. Self-management was encouraged through structured participation, supported by the provision of a *Brochure and Oral Care Calendar*. These educational tools likely contributed to the significant differences observed between the intervention and control groups. The findings of this study align with previous research, which has demonstrated the effectiveness of educational interventions in OM management among pediatric patients [13, 19, 20, 25]. Additionally, all children who completed the study received a certificate of appreciation, which served as a motivational tool, enhancing engagement and satisfaction with the intervention.

Following completion of the study, the oral mucositis care protocol is being considered for integration into routine nursing practice within the pediatric oncology unit. Implementation efforts include staff training, incorporation of the protocol into standard nursing care plans, and continued use of the educational brochure and oral care calendar. Future multi-center studies are needed to evaluate the feasibility and sustainability of implementing this protocol across different institutions and healthcare settings.

## Conclusion

In pediatric oncology clinics, the development and implementation of standardized oral care protocols are essential to enhance the quality of nursing care and mitigate the adverse effects of oral mucositis in children. Effective management requires a multidisciplinary approach, emphasizing collaboration among healthcare providers, the child, and their family. Engaging all stakeholders through tailored educational strategies is crucial to ensuring adherence and optimizing outcomes. As this study aimed to establish a standardized protocol, variations in the frequency or number of applications were not explored. Future research should focus on generating new evidence by evaluating the efficacy of different application frequencies and durations. Future studies should also incorporate additional clinical outcomes, such as adherence to chemotherapy, length of hospitalization, infection rates, body weight changes, and nutritional support requirements, to more comprehensively evaluate the impact of oral mucositis care protocols on overall clinical outcomes.

## Limitation

This study was conducted at a single center. Therefore, the responses may not be representative of the entire population who provide oral care to inpatients. In addition, since it is difficult to find patients who have not developed oral mucositis and since pediatric oncology is quite sensitive, the number of patients is limited. Another limitation is that, although MASCC/ISOO guidelines recommend daily oral assessment of oral mucositis, evaluations in this study were performed on Days 0, 3, 7, and 14 only due to clinical workload and feasibility constraints, which may have led to under-detection of short-term fluctuations in mucositis severity. Furthermore, randomization was not stratified according to key clinical variables such as chemotherapy duration or treatment intensity, which may have contributed to baseline imbalances between groups and potentially influenced mucositis risk. The study was also conducted in an unblinded manner at the level of participants and the nurse researcher delivering the intervention, and only the independent statistician performing the analyses was blinded to group allocation, which may introduce performance and detection bias. Finally, additional clinical outcomes (such as adherence to chemotherapy, length of hospitalization, use of rescue medications, infection status, body weight changes, and need for nutritional support) were not systematically collected, limiting our ability to link changes in mucositis severity with broader clinical outcomes.

## Data Availability

The datasets generated and/or analysed during the current study are not publicly available due to ethical restrictions and the protection of participant privacy, but are available from the corresponding author on reasonable request.
